# Illumina short read-based shotgun metagenomic data sets of simulated bacterial communities derived from fresh spinach and surface water

**DOI:** 10.1128/mra.00375-24

**Published:** 2024-06-11

**Authors:** Zhao Chen, Jianghong Meng

**Affiliations:** 1 Joint Institute for Food Safety and Applied Nutrition, and Center for Food Safety and Security Systems, University of Maryland, College Park, Maryland, USA; 2 Department of Nutrition and Food Science, University of Maryland, College Park, Maryland, USA; Montana State University, Bozeman, Montana, USA

**Keywords:** simulated community, Illumina sequencing, short read, shotgun metagenomics, sequencing depth, multidrug resistance, *Salmonella enterica*, *Pseudomonas aeruginosa*

## Abstract

Paired-end short reads of Illumina HiSeq, MiSeq, and NovaSeq of simulated bacterial communities from fresh spinach and surface water were generated *in silico* at various sequencing depths. Multidrug-resistant *Salmonella enterica* serotype Indiana was included in the spinach community, while the water community contained multidrug-resistant *Pseudomonas aeruginosa*.

## ANNOUNCEMENT

Simulated communities with precisely known relative abundances of each microorganism are commonly used to assess and validate metagenomic sequencing protocols and bioinformatic methodologies (1). Using the shotgun metagenomic data sets of Illumina short reads, representing simulated bacterial communities on fresh spinach and in surface water, we previously assessed the effectiveness of short-read assembly algorithms in Illumina sequencing to improve the metagenome-assembled genome-based identification of bacterial pathogens (2). Here, we introduce the data sets featuring simulated bacterial communities on fresh spinach and in surface water, which encompass *Salmonella enterica* serotype Indiana and *Pseudomonas aeruginosa*, respectively. Our data sets offer a meticulously crafted resource for researchers to benchmark the performance of Illumina sequencing in metagenomic analyses, particularly in identifying bacterial pathogens within complex food and environmental samples. By closely mimicking realistic scenarios, these data sets provide invaluable insights into the effectiveness of bioinformatic methodologies for metagenomic studies.

**Fig 1:**
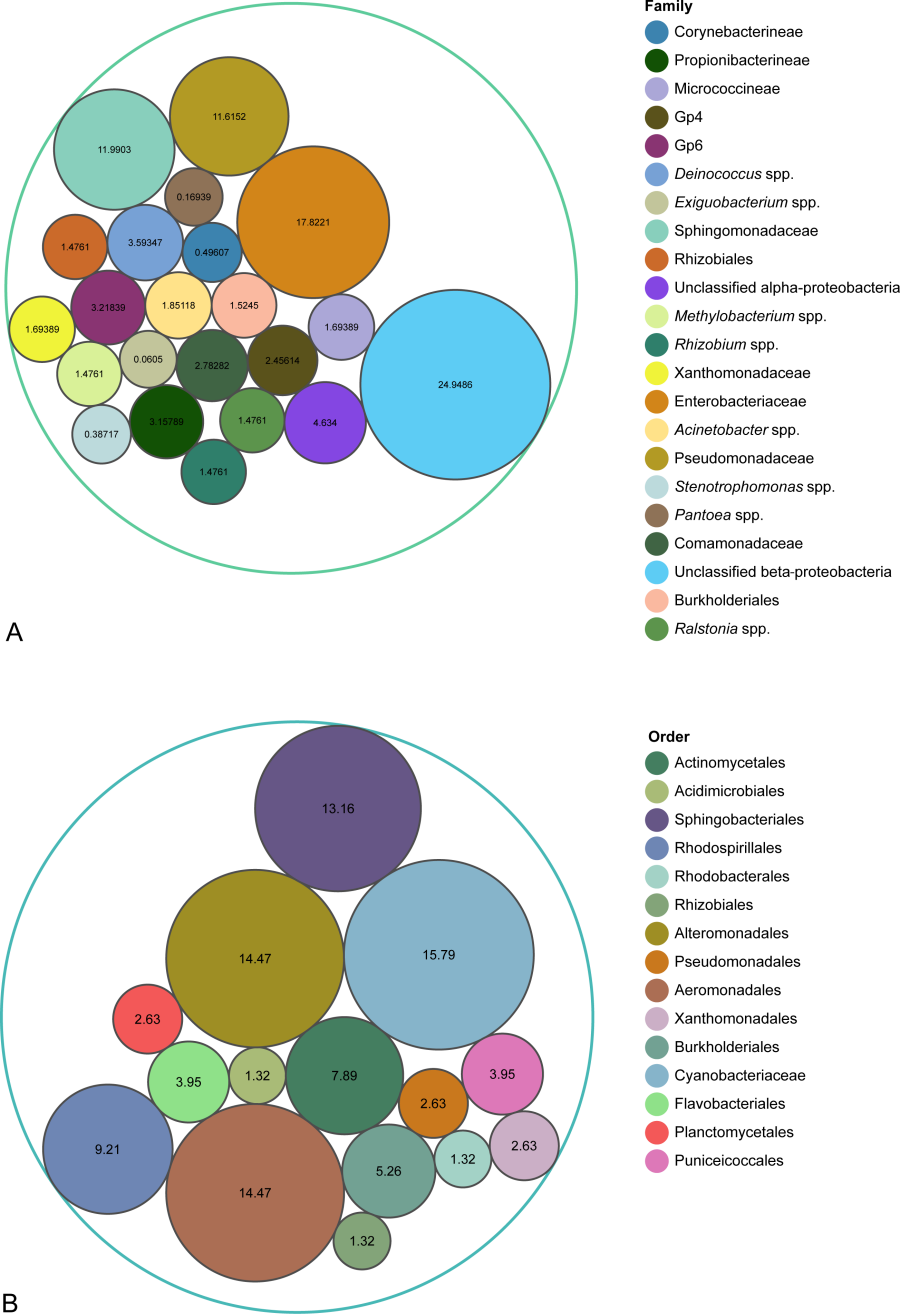
Relative abundance (%) of each family or order of the simulated bacterial communities on fresh spinach (**A**) and in surface water (**B**).

The community on fresh spinach was constructed using relative abundance data from Lopez-Velasco et al. (3), comprising five phyla: Acidobacteria, Actinobacteria, Deinococcus–Thermus, Firmicutes, and Proteobacteria (Alpha-, Beta-, and Gammaproteobacteria) (Fig. 1A; Table 1). Additionally, multidrug-resistant *S*. Indiana SI43 was incorporated to represent the Enterobacteriaceae family. In the community in surface water, formulated according to Beale et al. (4), six phyla were included: Acidobacteria, Actinobacteria, Bacteroidetes, Planctomycetes, Proteobacteria (Alpha-, Beta-, and Gammaproteobacteria), and Verrucomicrobia (Fig. 1B; Table 1). Multidrug-resistant *P. aeruginosa* PAO1 represented the Pseudomonadales order. In the original data used for simulation, while most reads were classified, some reads were unclassified or classified as eukaryotes. To ensure the fidelity of our data sets and focus on the bacterial communities of interest, the relative abundance was normalized to achieve a total of 100% for each bacterial community through a quality control step, with a specific emphasis on classified reads while excluding unclassified and eukaryotic reads.

**TABLE 1:** Representative microorganisms of the simulated bacterial communities on fresh spinach and in surface water

Community	Phylum	Family or order	Representative microorganism	GenBank assembly accession
Fresh spinach	Actinobacteria	Corynebacterineae	*Rhodococcus biphenylivorans* TG9	GCA_003288095.1
		Propionibacterineae	*Propionibacterium freudenreichii* FAM 14217	GCA_013205725.1
		Micrococcineae	*Micrococcus luteus* AS2	GCA_005280335.1
	Acidobacteria	Gp4	*Chloracidobacterium* sp. N	GCA_018304765.1
		Gp6	*Luteitalea pratensis* DSM 100886	GCA_001618865.1
	Deinococcus–Thermus	*Deinococcus* spp.	*Deinococcus ruber* JCM 31311	GCA_014648095.1
	Firmicutes	*Exiguobacterium* spp.	*Exiguobacterium acetylicum* AMCC 101217	GCA_008274845.1
	Alphaproteobacteria	Sphingomonadaceae	*Sphingomonas alpina* DSM 22537	GCA_009720245.1
		*Rhizobiales*	*Bradyrhizobium arachidis* CCBAU 051107	GCA_015291705.1
		Unclassified Alphaproteobacteria	*Brevundimonas diminuta* FDAARGOS 1026	GCA_016127655.1
		*Methylobacterium* spp.	*Methylobacterium oryzae* CBMB20	GCA_000757795.1
		*Rhizobium* spp.	*Rhizobium* sp. S41	GCA_001691455.1
	Gammaproteobacteria	Xanthomonadaceae	*Xanthomonas oryzae* pv. *oryzicola* YM15	GCA_001021915.1
		Enterobacteriaceae	*Salmonella enterica* serotype Indiana SI43	GCA_012053725.1
		*Acinetobacter* spp.	*Acinetobacter oleivorans* DR1	GCA_000196795.1
		Pseudomonadaceae	*Pseudomonas fluorescens* NCTC10038	GCA_900475215.1
		*Stenotrophomonas* spp.	*Stenotrophomonas maltophilia* NCTC10258	GCA_900475405.1
		*Pantoea* spp.	*Pantoea ananatis* LCFJ-001	GCA_016598655.1
	Betaproteobacteria	Comamonadaceae	*Diaphorobacter ruginosibacter* DSM 27467	GCA_014395975.1
		Unclassified Betaproteobacteria	*Massilia flava* DSM 26639	GCA_009789595.1
		Burkholderiales	*Rhizobacter gummiphilus* NBRC 109400	GCA_002762215.1
		*Ralstonia* spp.	*Ralstonia pickettii* FDAARGOS 410	GCA_002393485.1
Surface water	Actinobacteria	Actinomycetales	*Saccharopolysporaerythraea* NRRL 2338	GCA_000062885.1
		Acidimicrobiales	*Acidimicrobium ferrooxidans* DSM 10331	GCA_000023265.1
	Acidobacteria	Sphingobacteriales	*Sphingobacterium psychroaquaticum* SJ-25	GCA_004421025.1
	Alphaproteobacteria	Rhodospirillales	*Magnetospirillum gryphiswaldense* MSR-1	GCA_000513295.1
		Rhodobacterales	*Pikeienuella piscinae* RR4-56	GCA_011044155.1
		Rhizobiales	*Blastochloris viridis* ATCC 19567	GCA_001402875.1
	Gammaproteobacteria	Alteromonadales	*Alteromonas naphthalenivorans* SN2	GCA_000213655.1
		Pseudomonadales	*Pseudomonas aeruginosa* PAO1	GCA_000006765.1
		Aeromonadales	*Tolumonas auensis* DSM 9187	GCA_000023065.1
		Xanthomonadales	*Lysobacter caseinilyticus* KVB24	GCA_018406605.1
	Betaproteobacteria	Burkholderiales	*Pandoraea norimbergensis* DSM 11628	GCA_001465545.3
	Cyanobacteria	Cyanobacteriaceae	*Cyanobacterium aponinum* PCC 10605	GCA_000317675.1
	Bacteroidetes	Flavobacteriales	*Gramella flava* JLT2011	GCA_001951155.1
	Planctomycetes	Planctomycetales	*Planctopirus ephydatiae* spb1	GCA_007752345.1
	Verrucomicrobia	Puniceicoccales	*Coraliomargarita akajimensis* DSM 45221	GCA_000025905.1

Paired-end short reads were simulated using InSilicoSeq 1.5.4 (5) with default settings based on pre-computed error models derived from three Illumina sequencing platforms, including HiSeq (2 × 125 bp), MiSeq (2 × 300 bp), and NovaSeq (2 × 150 bp). The MiSeq model was developed using shotgun metagenomic data sets obtained from an ocean water sample collected in Kenya (run accession: ERR1912174), and those sourced from human samples on Illumina Basespace were utilized in building the HiSeq and NovaSeq models (5). One million reads of each sequencing platform were generated independently for the fresh spinach community, with relative abundance drawn from a log-normal distribution. Additionally, 2.4 million HiSeq and 2 million NovaSeq reads were produced to ensure all sequencing platforms had the same sequencing depth. The number of MiSeq reads was increased to 1.5 million. For the surface water community, 2.4 million HiSeq, 1.5 million MiSeq, and 2 million NovaSeq reads were initially simulated. Then, to achieve uniform sequencing depth across platforms, the numbers of HiSeq, MiSeq, and NovaSeq reads were increased to 4.8, 2, and 4 million, respectively.

Through meticulous simulation, we have ensured that our data sets faithfully mirror real-world conditions, thereby offering insights into the compositions of bacterial populations in diverse microbiomes.

## Data Availability

Simulated shotgun metagenomic data sets have been deposited into the Sequence Read Archive (SRA) database on National Center for Biotechnology Information (NCBI) under the BioProject accession number PRJNA1090476.
